# Defective Drainage

**Published:** 1874-05

**Authors:** S. J. Parker


### Defective Drainage.

In the growth of every town and State there is always a point reached where it needs all the appliances for its convenience and sanitary condition that art and experience can give. And that too when its pecuniary ability is least fitted to or prepared to meet them. Such is our own condition. With an University of the noblest proportions, struggling upwards in its ample dimensions, attempting to reach and pass in ten years the growth of centuries, with a sudden inflow of a large population, with the task of remodelling our public schools requiring large expenditures—with our five railroads, all but one or two built unwisely by the operation of the bonding of towns by the late unfortunate law, that puts us and our posterity in debts, oppressive and wrong; with changes in our dwellings to meet modern taste; with the libraries and other buildings, enlargement of stocks and stores for business; with the advance of the whole cost of material, labor to an extravagant degree,—one and all would seem to make this the last and most unfavorable time for the Committee of Physicians of Ithaca to try still further the burdens that a proper sanitary condition of our town imposes on us.

While, therefore, the malaria of our town is mild in its manifestations as compared with that of many other places, it neversurely causes malarious cachexia with its train of disorders--dyspepsia, neuralgia, rheumatism, and the like, in general prevalence of ill health, for the relief of which we advocate the lowering of the lake, as it may be the slow but sure means of putting an end to the miasm by the draining and drying of the flat, marshy lands which are now subject to inundations. And in this I am in perfect accord with my colleague in this Committee Report, that it is the never-ceasing dampness, the constant presence of water in the wet portions of our town, that is the cause of our miasm. At least one-third of our dwellings at present are on lots underlaid by clay, or on clayish sand, but little removed from the permanent water level; and, because of the cheapness of the building lots on these sites, houses are being multiplied and approximating nearer and nearer to the water level. Twenty years ago, we thought three feet above permanent water level too little. Many dwellings are to-day not more than twelve to fifteen inches above the water level, and some are within a spade's depth. And the occupants of these houses, men, women, and children, who are rarely or never well, fail to recognize and deny the cause of their ill-health.

We must awake to our own health. We must rouse the slumbering citizens of other populous neighborhoods to our common interest. We must reach the intelligence of the whole State on this question. Reason, health and public good are on our side. For our own Ithaca, it is a home to be proud of, except in this one crying evil.—*S. J. Parker, M.D., Sanitarian for June.*

